# A Rare Case of Hypoparathyroidism and Myxedema Coma in a Patient With Diamond-Blackfan Anemia

**DOI:** 10.7759/cureus.19941

**Published:** 2021-11-27

**Authors:** Raffaele A Ruggiero, Qasim Z Iqbal, Ali Akram, Jared Dendy, Julie Zaidan

**Affiliations:** 1 Internal Medicine, Northwell Health, New York, USA; 2 Internal Medicine, Ochsner Clinic Foundation, New Orleans, USA; 3 Internal Medicine, Lahore Medical and Dental College, Lahore, PAK; 4 Internal Medicine, Wright Center, Scranton, USA; 5 Endocrinology, Diabetes, and Metabolism, Ochsner Clinic Foundation, New Orleans, USA

**Keywords:** internal medicine and endocrinology, blood transfusion safety, myxedema coma, diamond-blackfan anemia, hypothyroidism

## Abstract

Diamond-Blackfan anemia (DBA) is a rare genetic condition that presents due to bone marrow failure caused by a dysfunction in ribosomal biogenesis and function. The patients would often require chronic transfusions as treatment, which puts them at high risk for the development of secondary hemochromatosis. This secondary hemochromatosis results in endocrinopathies due to iron deposition into the endocrine glands. We present an interesting case report of a female patient with multiple endocrinopathies due to secondary hemochromatosis resulting from chronic transfusion therapy. Her endocrinopathies included hypothyroidism complicated by myxedema coma and, interestingly, hypoparathyroidism, which has seldom been reported in DBA patients. Early diagnosis and precise treatment of life-threatening conditions like myxedema coma in DBA patients can avoid morbidity and mortality.

## Introduction

Diamond-Blackfan anemia (DBA) is a rare genetic condition that results from bone marrow failure, due to a dysfunction in ribosomal biogenesis and function. The patients of this condition need to undergo chronic transfusions as treatment, which puts them at high risk for the development of secondary hemochromatosis. This secondary hemochromatosis leads to endocrinopathies because of iron deposition into the endocrine glands. This deposition of iron causes malfunction of endocrine glands causing the patient to suffer from multiple endocrinopathies.

## Case presentation

A 22-year-old female with a pertinent medical history of DBA and chronic hepatic thrombosis (on anticoagulation with Eliquis) was admitted to the hospital for left upper back cellulitis. Unfortunately due to her anemia (baseline hemoglobin: 9 g/dl), she had required transfusions with packed red blood cells every three to four weeks and was also on daily chronic chelation therapy with deferasirox; her last transfusion had been four days before the admission. Her history was further complicated by multiple endocrinopathies including hypothyroidism, hypogonadism, growth hormone deficiency, and insulin-dependent diabetes mellitus. On admission, the patient's vital signs were normal. Her initial blood work included a metabolic panel (Table 1) revealing hyperglycemia, hypocalcemia, high phosphorus, and transaminitis. Findings on a CT scan of the chest correlated with cellulitis overlying the left trapezius muscle for which empiric treatment with broad-spectrum IV antibiotics was initiated. She was subsequently started on subcutaneous weight-based insulin for hyperglycemia, calcium supplementation through calcium drip at 50 cc/hour of calcium gluconate for 24 hours; an electrocardiogram was done, which revealed normal sinus rhythm, and an ultrasound of the liver was performed, but it revealed no obvious abnormalities, deeming her transaminitis likely a result of chronic hepatic thrombosis combined with hepatic iron deposits from chronic transfusions.

**Table 1:** Metabolic panel AST: aspartate aminotransferase; ALT: alanine aminotransferase; GFR: glomerular filtration rate

Variables	Reference range	Patient value
Calcium	8.7–10.5 mg/dL	6.8
Phosphorus	2.1–4.9 mg/dL	9.7
Albumin	3.4–5.4 g/dl	4.2
AST	10–40 u/l	114
ALT	4–36 u/l	118
Bicarbonate	22–28 meq/l	21
Hemoglobin A1c	4–5.6%	9.8
Glucose	Less than 100 mg/dl	428
Anion gap	3–10 meq/l	7
Creatinine	0.6–1.2 mg/dl	1.2
GFR	Greater than 60	>60

On day two of her hospitalization, a rapid response was called on the patient as she fell backward from a sitting position and became unresponsive. She was placed on a monitor, which revealed sinus bradycardia with a heart rate of 36 bpm and hypotension with a blood pressure of 68/40 mmHg. Her pulse oximeter was 100% on a non-rebreather. TPA was held due to her being on anticoagulation. Within a few minutes, she became responsive but revealed poor motor response on physical exam with diminished reflexes. She was taken for a CT scan of the head along with a CT perfusion study of the head, but both revealed no acute findings. Blood work was performed, which included tests for thyroid-stimulating hormone (TSH), free thyroxine (FT4), T3, complete blood count (CBC), blood cultures, an 8-am cortisol level, and a metabolic profile. On the morning of hospital day three, the patient became unresponsive again. Vital signs were again significant for bradycardia and hypotension. She remained unresponsive for a few minutes and then quickly returned to baseline. Her physical exam persistently revealed diminished reflexes bilaterally. The patient was managed for possible seizure activity and was loaded with levetiracetam, and an electroencephalogram (EEG) was performed, which revealed normal results. Later in the day, her laboratory results for thyroid function came back and were significant for an elevated TSH and a suppressed FT4 (Table 2).

**Table 2:** Thyroid function test TSH: thyroid-stimulating hormone

Variables	Reference range	Patient value
TSH	0.400–4.000 mIU/mL	288
Free T4	0.71–1.9 ng/dL	<0.1
T3	80–200 ng/dl	<20

The patient's history was taken again by the covering physician, and she revealed a history of hypothyroidism along with non-compliance with her home dose of 100 mcg of levothyroxine. On further review of her medical records, it was found that she was negative for anti-thyroid peroxidase antibody, and an ultrasound of the thyroid gland from last year was non-revealing of any thyroid nodules. She was seen by endocrinology, who attributed her condition to myxedema coma. The patient did not have a baseline 8-am cortisol level result, and hence she was given a stress dose of 100 mg of IV hydrocortisone and started on a standing dose of 100 mg hydrocortisone every eight hours. She was then given a bolus dose of 400 mcg IV levothyroxine, put back on her home dose of oral levothyroxine, and started on triiodothyronine 5 mcg twice daily. She improved clinically in the next three days and her morning cortisol drawn before treatment was initiated came out to be normal (16 mcg/dl). Her triiodothyronine, antiepileptic medication, and hydrocortisone were gradually tapered off. Her blood cultures came out positive for methicillin-sensitive *Staphylococcus aureus* and she completed a course of antibiotics for the underlying cellulitis. Given her studies were significant for hypocalcemia and elevated phosphorus, parathyroid hormone (PTH) was checked during her hospitalization. The PTH results showed a suppressed intact PTH (Table 3) and confirmed our suspicion of hypoparathyroidism in her. After initially being placed on a drip of calcium gluconate, she was started on daily oral calcium carbonate, which was asked to continue on discharge. She was also started on calcitriol 0.5 mcg daily. She was advised on the importance of compliance with her medications and to have a close follow-up with her endocrinologist on discharge.

**Table 3:** Laboratory results

Variables	Reference range	Patient value
Calcium	8.7–10.5 mg/dL	6.8
Phosphorus	2.1–4.9 mg/dL	9.7
Albumin	3.4–5.4 g/dl	4.2
Vitamin D, 25 OH	30–80 ng/mL	8
Vitamin D, 1,25 OH	19.9–79.3 ng/mL	5
Parathyroid hormone	15–65 pg/ml	2

## Discussion

DBA is an extremely rare syndrome, which affects five to seven per million live births per year [1]. It was recognized as a distinct clinical entity in 1938 and classified as one of the rare inherited bone marrow failure syndromes [2]. The syndrome is a result of a cellular defect in which erythroid progenitors and precursors are highly sensitive to death by apoptosis leading to an erythropoietic failure. It is characterized by bone marrow failure, congenital anomalies, and predisposition to cancer. A variety of congenital anomalies including craniofacial, ophthalmologic, urogenital, cardiac, and neuromuscular have been reported. Craniofacial is the most common, involving 50% of all reported congenital anomalies. DBA is believed to be a disorder of ribosome biogenesis and function with 50% of patients having a single gene mutation in the gene encoding a ribosomal protein. The most common mutation reported involves RPS19 [3]. It is mainly inherited through an autosomal-dominant pattern, but there have been cases with sporadic or different patterns of familial inheritance.

The initial treatment of DBA begins at the age of one with corticosteroids. Prednisone is started at a dose of 2 mg/kg/day and gradually reduced to ≤0.5 mg/kg/day. Roughly 80% of patients respond to steroids given as the initial course, but only 40% have a sustained response without dose-limiting toxicity. The chronic use of corticosteroids therapy predisposes these patients to iatrogenic Cushing's syndrome and adrenal insufficiency. Chronic transfusion therapy is usually initiated in DBA patients once it has been established that they are no longer responsive to corticosteroids. Importantly though, if chronic transfusion therapy is initiated, the corticosteroid regimen is tapered off and discontinued. Unfortunately, around 40% of these patients become transfusion-dependent, requiring packed red blood cells every three to four weeks [1]. Furthermore, on reviewing the literature, the role of hematopoietic stem cell transplant remains controversial in these patients. The use of chronic blood transfusions places DBA patients at risk for secondary hemochromatosis, which can lead to a variety of different complications. About 23% of all deaths reported to the DBA American Registry are related to complications from iron overload [4]. It has been found in the literature that around 20% of patients enter remission and require neither corticosteroids nor transfusions for longer than six months.

Endocrinopathies can develop as a result of direct iron deposition into endocrine glands and have been reported in a few studies [5]. A French cohort study revealed that 14% of DBA patients developed iron-related endocrine or other organ complications, and an Italian cohort study revealed that 23% of DBA patients developed the same [1,5,6]. To help minimize these complications, chelation therapy is often initiated. However, despite chelation therapy, complications from iron overload and endocrinopathies are still reported in these patients [7,8]. Reviewing a report from the DBA Registry, it was seen that 53% of DBA patients were found to have one or more endocrine disorders present in them. Of note, 45% of chronically transfused patients had endocrinopathies with the most common being adrenal insufficiency. This adrenal insufficiency was noted more in the patients receiving chronic glucocorticoid treatment. Hypogonadism and hypothyroidism were the second and third most common endocrinopathies seen in these patients.

Our patient had multiple endocrinopathies including hypothyroidism, hypogonadism, growth hormone deficiency, diabetes mellitus, and, interestingly, hypoparathyroidism. All these findings were likely due to iron overload from chronic transfusion therapy. Findings of increased ferritin level and transaminitis secondary to iron deposit were promising clues to her being iron-overloaded. Conversely, studies have shown that the correlation between liver iron concentration and ferritin is not strong. Consequently, a single ferritin measurement is a poor predictor of liver iron concentration. Our patient had ferritin levels checked multiple times, and a liver biopsy was done by the hepatology team, confirming the suspicion of secondary hemochromatosis.

The patient had been poorly compliant with her hypothyroidism medications and subsequently presented with the life-threatening complication of myxedema coma. This condition represents a decompensated state of severe untreated hypothyroidism where the body is unable to maintain homeostasis by neurovascular adaptations essential for hemodynamic stability [9]. It is not a common condition and has an incidence of 22 per million per year; however, it has a very high mortality rate of up to 20-25% [10]. Myxedema coma is usually associated with various precipitating factors, such as the infection seen in our patient’s case. The cardinal manifestation is a deterioration of mental status. Our patient had several clinical manifestations, which are common in myxedema coma, such as diminished cognitive status, lethargy, somnolence, bradycardia, hyporeflexia, and hypotension. Hypothermia was not seen in our patient; however, it has been reported in the literature that patients do present as afebrile during an active infection [11].

Another interesting physical presentation in our patient was of facial puffiness and periorbital edema; both are common findings in myxedema coma [12]. There are no specific diagnostic criteria for myxedema coma, but diagnosis is generally made with a combination of lab findings and clinical manifestations [13]. Patients usually have a high TSH with a low T4 and T3. Our patient had an extremely elevated TSH with a low fT4 and T3. The central tenants of treatment revolve around thyroid hormone replacement and supportive care [13]. Supportive care including IV fluids, electrolyte replacement, and warming blankets are also done. Optimum treatment with thyroid hormone replacement is still uncertain due to the lack of clinical trials [10]. In general, patients are treated with levothyroxine 200 to 400 mcg IV, followed by daily doses of 50 to 100 mcg, and triiodothyronine 5 to 20 mcg IV, followed by 2.5 to 10 mcg every eight hours. The lower end of the dose ranges is preferred in lower-weight and older patients and those at risk for cardiac complications. Hydrocortisone 100 mg IV every eight hours is also given until the exclusion of possible adrenal insufficiency.

Hypoparathyroidism is an extremely rare complication of transfusion-dependent patients. It occurs as a result of iron deposition into the parathyroid gland. The dysfunction of the parathyroid leads to deficient secretion of PTH, resulting in absent signaling in classic target tissues [14]. In the study done by the DBA Registry, there were no patients diagnosed with hypoparathyroidism; however, there was heavy suspicion in two patients [1]. Hypoparathyroidism, though, has been reported in patients with transfusion-dependent thalassemia as well as in a French cohort study on DBA patients [6]. Diagnosis can be made with low serum ionized or albumin correct calcium concentration with a low or undetectable PTH. Our patient had a low PTH with low corrected calcium, which was consistent with a diagnosis of hypoparathyroidism.

## Conclusions

DBA is a rare genetic condition occurring due to marrow failure. The patients are often required to undergo chronic transfusions for treatment, which puts them at high risk for the development of secondary hemochromatosis. Our patient presented with hypoparathyroidism, which has not been reported in a DBA patient before, based on our elaborative review of the literature. Early diagnosis and precise treatment of life-threatening conditions like myxedema coma and hypoparathyroidism in DBA patients can help us save their lives.

## References

[1] Lahoti A, Harris YT, Speiser PW, Atsidaftos E, Lipton JM, Vlachos A (2016). Endocrine dysfunction in Diamond-Blackfan anemia (DBA): a report from the DBA Registry (DBAR). Pediatr Blood Cancer.

[2] Vlachos A, Blanc L, Lipton JM (2014). Diamond Blackfan anemia: a model for the translational approach to understanding human disease. Expert Rev Hematol.

[3] Vlachos A, Ball S, Dahl N (2008). Diagnosing and treating Diamond Blackfan anaemia: results of an international clinical consensus conference. Br J Haematol.

[4] Roggero S, Quarello P, Vinciguerra T, Longo F, Piga A, Ramenghi U (2009). Severe iron overload in Blackfan-Diamond anemia: a case-control study. Am J Hematol.

[5] Al-Agha AE, Bawahab NS, Nagadi SA, Alghamdi SA, Felemban DA, Milyani AA (2020). Endocrinopathies complicating transfusion-dependent hemoglobinopathy. Saudi Med J.

[6] Chen S, Warszawski J, Bader-Meunier B, Tchernia G, Da Costa L, Marie I, Dommergues JP (2005). Diamond-blackfan anemia and growth status: the French registry. J Pediatr.

[7] Lanes R, Muller A, Palacios A (2000). Multiple endocrine abnormalities in a child with Blackfan-Diamond anemia and hemochromatosis. Significant improvement of growth velocity and predicted adult height following growth hormone treatment despite liver damage. J Pediatr Endocrinol Metab.

[8] Willig TN, Niemeyer CM, Leblanc T (1999). Identification of new prognosis factors from the clinical and epidemiologic analysis of a registry of 229 Diamond-Blackfan anemia patients. DBA group of Société d'Hématologie et d'Immunologie Pédiatrique (SHIP), Gesellshaft für Pädiatrische Onkologie und Hämatologie (GPOH), and the European Society for Pediatric Hematology and Immunology (ESPHI). Pediatr Res.

[9] Kwaku MP, Burman KD (2007). Myxedema coma. J Intensive Care Med.

[10] Klubo-Gwiezdzinska J, Wartofsky L (2012). Thyroid emergencies. Med Clin North Am.

[11] Wiersinga WM (2000). Myxedema and coma (severe hypothyroidism). Endotext.

[12] Wall CR (2000). Myxedema coma: diagnosis and treatment. Am Fam Physician.

[13] Leung AM (2016). Thyroid Emergencies. J Infus Nurs.

[14] Shoback DM, Bilezikian JP, Costa AG (2016). Presentation of hypoparathyroidism: etiologies and clinical features. J Clin Endocrinol Metab.

